# rAAV Fbxo7 gene therapy rescues the progressive nigrostriatal pathology in a mouse model of juvenile parkinsonism

**DOI:** 10.1186/s40478-026-02294-y

**Published:** 2026-04-16

**Authors:** Sara Al Rawi, Pamela Tyers, Roger A. Barker, Heike Laman

**Affiliations:** 1 Department of Pathology, University of Cambridge, Tennis Court Road, Cambridge, CB2 1QP UK; 2 Department of Clinical Neuroscience, University of Cambridge, John Van Geest Centre for Brain Repair, Cambridge, UK; 3 Stem Cell Institute, University of Cambridge, Jeffrey Cheah Biomedical Centre, Cambridge, CB2 0AW UK

**Keywords:** Fbxo7, Parkinson’s disease, Gene therapy, Striatum, Mouse model

## Abstract

Mutations in the *PARK15/FBXO7* gene cause an early-onset parkinsonian syndrome. A mouse with conditional knockout of Fbxo7 in dopaminergic neurons models key features of this pathology, exhibiting a progressive loss of striatal tyrosine hydroxylase (TH)-positive terminals that precedes cell death. This gradual decline in a mammalian model provided an opportunity to test the capacity of Fbxo7 to act as a therapeutic agent and define a treatment window for gene replacement. Using a recombinant adeno-associated virus (rAAV), we delivered Fbxo7 to test its capacity to prevent (delivery prior to deficit onset) and rescue (delivery after nigrostriatal phenotypes were evident) dopaminergic markers. Remarkably, Fbxo7 re-expression rescued DAT and TH-immunoreactivity in the striatum and nucleus accumbens before and after the onset of the loss of TH + staining. These data establish that Fbxo7-dependent neurodegeneration is not an irreversible process, highlighting its regulated pathways as promising potential targets for developing therapies that will not only slow disease progression, but also ameliorate dopaminergic terminal deficits and potentially restore function.

## Introduction

While most cases of Parkinson’s disease (PD) arise sporadically in individuals over 50, rare, early-onset genetic forms provide an invaluable insight into the underpinning mechanisms of the disease. These monogenic forms often disrupt fundamental cellular systems, like autophagy, mitochondrial function, and proteostasis [1–3]. Crucially, the dysfunction of these same pathways is strongly implicated in sporadic PD, highlighting the wider relevance of insights gained from these inherited forms of disease. These systems are deeply interconnected, and dysfunction in one, such as protein homeostasis, can trigger a cascade of exacerbating dysfunction in others, accelerating disease progression.

To dissect these mechanisms, our group previously developed a murine model with conditional knockout (cKO) of the *FBXO7* gene in dopaminergic (DA) neurons [4], and this mouse recapitulates key features of human PD. It exhibits a progressive nigrostriatal degeneration that begins with a reduction in cell body size of DA neurons in the substantia nigra pars compacta (SNpc), followed by a marked loss of striatal tyrosine hydroxylase (TH) + terminals and dopamine levels in the striatum by 6 weeks, and culminates in significant SNpc cell death by 20 weeks and motor deficits at 30 weeks. Our study, alongside several other mouse models of conditional Fbxo7 loss, establishes its essential neuroprotective role [4–7].

Fbxo7 is a multi-functional E3 ubiquitin ligase receptor subunit implicated in a number of cellular pathways affecting many disparate tissues, including neurons but also highly proliferative cell types, like T cells, red blood cells and spermatocytes [8]. Mechanistically relevant to the neurodegenerative processes in PD, these cellular processes include mitophagy and proteostasis [1, 9–11]. Mutations in Fbxo7 cause a parkinsonian syndrome in patients that can range from infantile to adult onset and be clinically indistinguishable from idiopathic PD cases [11–27]. This makes investigations into Fbxo7-driven neurodegeneration biologically relevant for understanding the aetiology of sporadic PD. The progressive decline observed in our cKO mouse model presented us with an opportunity to address a key therapeutic question: is the neurodegeneration driven by Fbxo7 loss reversible? We hypothesised that directly restoring Fbxo7 expression would provide a treatment. To address this, we used recombinant adeno-associated virus (rAAV) to restore Fbxo7 expression in our mouse at two distinct stages: prophylactically before the onset of phenotypes, and as a rescue therapy after the striatal phenotype was established. We found that rAAV-mediated Fbxo7 expression, delivered either before or after the onset of the striatal deficit, robustly restored DAT and TH immunoreactivity. These findings demonstrate that the neurodegeneration driven by Fbxo7 dysfunction may not only be preventable but also reversible. This positions Fbxo7-regulated pathways as promising targets for developing restorative, rather than merely palliative, therapies for PD.

## Results

### Prophylactic Fbxo7 expression prevents the onset of nigrostriatal degeneration

**Fig. 1:**
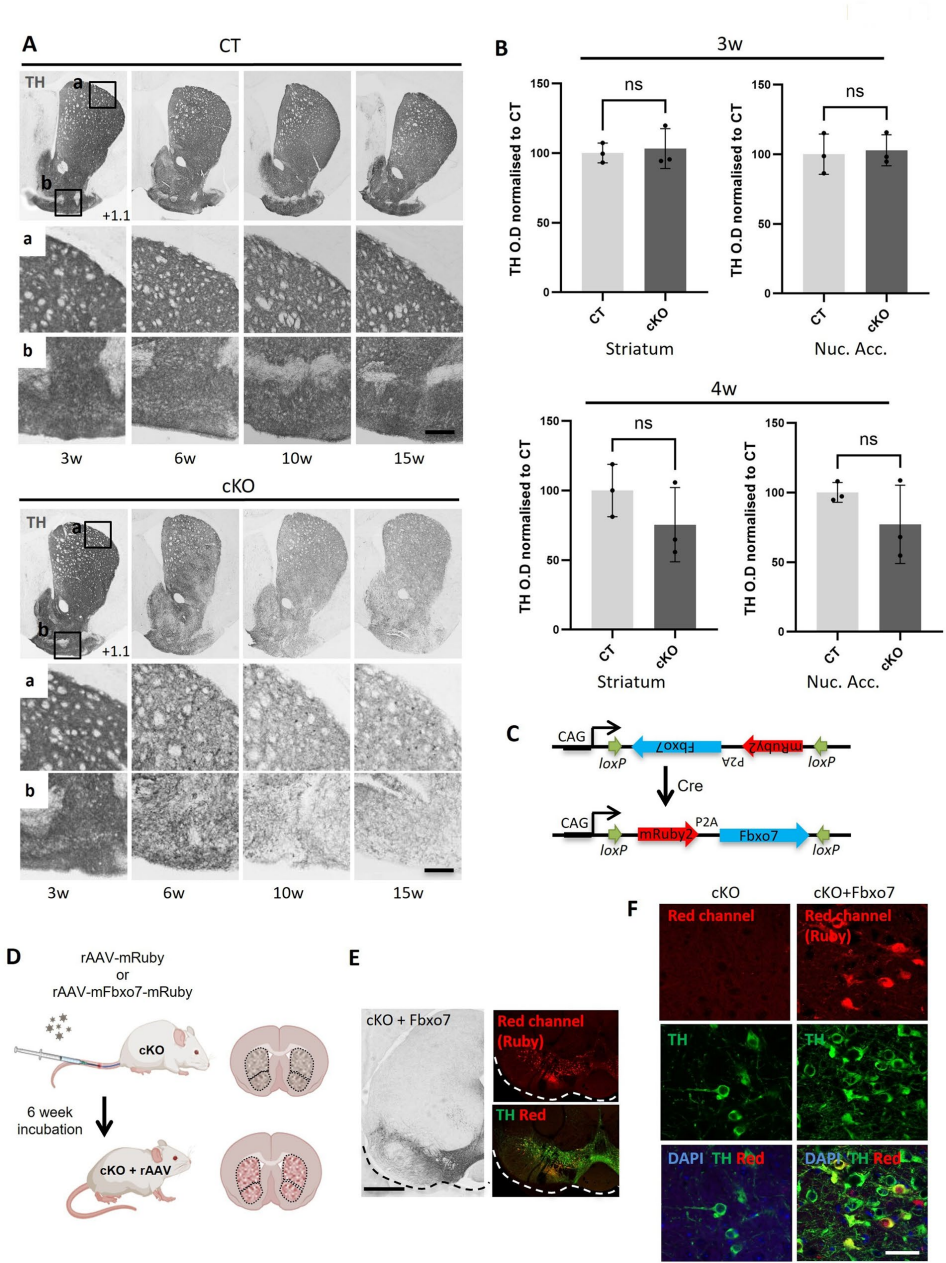
**A** TH-immunostained sections of the striatum at + 1.1 mm relative to bregma from control (CT; Fbxo7fl/−) and conditional KO (cKO; DatCre Fbxo7fl/−) mice at the indicated ages. (**a**, **b**) Magnified boxed regions showing TH + fibres in the striatum (a) and olfactory tubercle (**b**). Scale bars: 800 μm (top panels), 300 μm (**a**, **b**). **B** Optical density (OD) quantification of TH + fibre density in CT and cKO mice at 3 and 4 weeks of age. Values are normalised to the mean of control (CT; Fbxo7fl/−) and are shown as mean ± SEM with individual data points. n = 3 per group. Statistical comparisons were performed using unpaired two-tailed t-tests. ns, not significant (p ≥ 0.05). **C** Schematic map of rAAV transgenes and their orientation after transduction into dopaminergic neurons expressing Cre. **D** Gene therapy protocol schematic. Image created with BioRender. **E** TH immunostaining in the substantia nigra pars compacta (SNpc) from cKO (cKO; DatCre Fbxo7fl/−) mice injected with Fbxo7 virus. DAB staining (dark grey, left panel) and Alexa Fluor 488 (green, lower right panel) are shown. mRuby expression is shown in red. Scale bar: 500μm **F** Magnified view of TH + neurons in the SNpc (TH, Alexa Fluor 488, green). No red signal is observed in uninjected cKO mice, while mRuby expression is observed in Fbxo7 virus-injected cKO mice. Scale bars: 30μm

We previously reported that beginning around 6 weeks of age, *Dat*^*Cre*^* Fbxo7*^*fl/−*^ mice, referred hereafter as conditional KO (cKO) mice, exhibit progressive phenotypes resulting from the loss of Fbxo7 expression in dopamine (DA) neurons. These phenotypes include a reduction in striatal dopamine release, loss of dopaminergic terminals in the striatum and eventual loss of TH + cell bodies in the SNpc by 20 weeks of age [4]. For the purposes of this study, we chose TH immunostaining as a biomarker since it is the earliest marker of pathological change in cKO mice. Specifically, *Fbxo7*^*fl/−*^ mice, hereafter referred to as control (CT) mice, show robust and uniform TH immunostaining across the striatum. In contrast, cKO mice display a significant reduction in TH + terminal density in the striatum and nucleus accumbens at 6 weeks of age (Fig. 1A). At 12 weeks, striatal TH immunoreactivity has plateaued in CT mice, while cKO mice continue to show a significant reduction in TH + terminal density at 10 and 15 weeks (Fig. 1A). To test if early Fbxo7 re-expression could prevent this phenotype of early TH + terminal loss, we first identified an optimal therapeutic window for intervention. We previously reported normal striosome formation at P0, and no obvious deviation of TH + fibres along the medial forebrain bundle in cKO mice [4]. Quantification of TH + terminals density confirmed that at 3 weeks of age, cKO mice were indistinguishable from CT mice. By 4 weeks, a trend toward reduced TH + terminal density was apparent but remained non-significant, marking this as an appropriate timepoint, prior to the emergence of any significant deficit, for intervention (Fig. 1B). We chose rAAV-mediated gene delivery, selecting the AAV9 serotype that can cross the blood–brain-barrier specifically in C57BL/6 mice, making it possible to deliver viruses intravenously rather than by stereotactic injection into neonates [28]. In addition to refining the delivery method for the mice, this approach also offered the advantage of allowing mice to develop normally and wean, so that juvenile and adult mice could be treated. We engineered Cre-dependent rAAV constructs using a double floxed inverted open reading frame (DIO, 'flexed') strategy. Upon recombination, the constructs expressed mRuby as a fluorescent tracer of expression either alone (empty vector, EV) or in combination with murine Fbxo7, with bicistronic expression enabled by a P2A ribosomal skipping sequence. This construct allows transgene expression specifically in Cre recombinase-expressing cells (Fig. 1C). At 4 weeks of age, mice received a single intravenous inoculum of either the virus containing Fbxo7 and mRuby (Fbxo7 virus) or 3.7 times more virus expressing mRuby only (EV virus) (see Materials & Methods) and were left to recover for 6 weeks to allow them to fully express the transgene in vivo prior to harvest (Fig. 1D). Brain sections were processed for immunohistochemistry, with mRuby fluorescence directly visualised and quantified, and TH expression analysed by DAB staining. Expression of mRuby was evident and restricted to the midbrains of injected mice (Fig. 1E). Moreover, this fluorescence was specific to the target population as evidenced by clear, restricted expression within TH + neurons of the SNpc (Fig. 1E, F). Transduction efficiency, quantified as the percentage of TH + cells positive for mRuby, was over 61.4 ± 10.4% (mean ± SD) for the Fbxo7 virus group and 82.7 ± 13.6% (mean ± SD) for the EV virus group (n = 3), confirming transgene expression in TH + neurons.

**Fig. 2:**
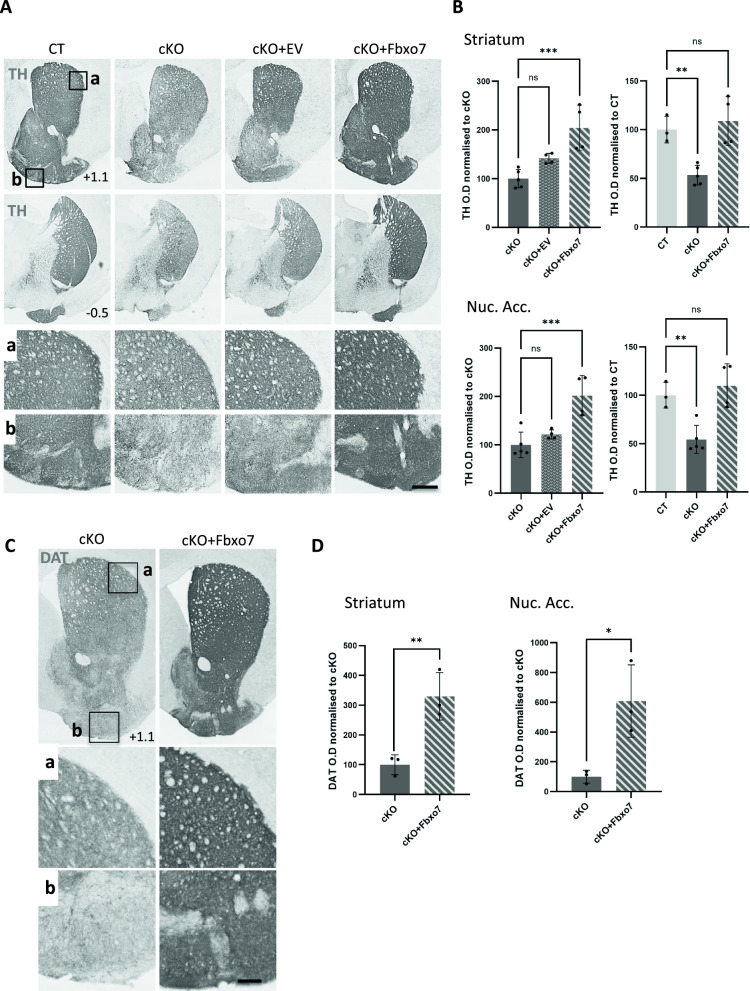
**A ** Images of TH-immunostained striatum at + 1.1 mm (top) and − 0.5 mm (bottom) relative to bregma are shown. **B ** OD quantification of TH + fibre density in CT (*Fbxo7*^*fl/−*^) (n = 3), cKO (*Dat*^*Cre*^* Fbxo7*^*fl/−*^) (n = 5), and cKO mice injected with rAAVs (EV or Fbxo7, as indicated) at 4 weeks and harvested at 10 weeks (n = 4). Values are normalised to cKO or CT (as indicated on the y axis) and are presented as mean ± SEM with individual points. Statistical analysis was performed using one-way ANOVA followed by Šídák's correction as post-hoc tests comparing each group to cKO or CT. (**a**,** b**) Magnified boxed regions of striatum (**a**) and olfactory tubercle (**b**). Scale bars: 800 μm (upper panels), 300 μm (lower a, b panels). **p < 0.01; ***p < 0.001; ns, not significant **C ** Images of DAT-immunostained striatum at + 1.1 mm relative to bregma. (**a**,** b**) Magnified boxed regions of striatum (**a**) and olfactory tubercle (**b**). **D ** OD quantification of DAT + fibre density in cKO mice at 10 weeks, uninjected or injected with Fbxo7 virus. Values are normalised to the cKO mean and presented as mean ± SEM with individual points. n = 3 for both groups. Statistical comparisons were performed using unpaired two-tailed t-test. Scale bars: Panel A, C: 800 μm (top panel), 300 μm (**a**,** b**)

We then wanted to test the capacity of rAAV delivery of Fbxo7 to act as a treatment in 4-week-old cKO mice to modulate the TH levels as a biomarker of emerging nigrostriatal pathology. To quantify striatal TH + staining, brain sections were processed for immunohistochemistry and visualised using diaminobenzidine (DAB) staining (Fig. 2A). Mean optical density across the dorsal striatum (*Striatum*) and the nucleus accumbens (*Nuc.Acc*.) was measured on TH-stained sections and used to determine the density of TH + terminals (see Materials & Methods) (Fig. 2B). TH signal from brain sections from cKO mice injected with EV virus and Fbxo7 virus was quantified as a percentage relative to untreated cKO mice (cKO ≈ 100%). Injection of EV virus into cKO mice produced a modest non-significant increase in TH + signal in the striatum (141%, p = 0.19) and nucleus accumbens (121%, p = 0.73). In contrast, treatment of cKO mice by injection with Fbxo7 virus significantly increased TH + signal to 204% in the striatum (p = 0.0006) and 202% in the nucleus accumbens (p = 0.0006). At 10 weeks of age untreated cKO mice exhibit a significant reduction in TH + signal, with a 46.8% decrease in the dorsal striatum (p = 0.0057) and a 45.5% decrease in the nucleus accumbens (p = 0.0071) compared to CT mice. Notably, treatment of cKO mice with Fbxo7 virus resulted in TH + staining to levels comparable to CT mice at 10 weeks of age (~ 110% of CT levels in both the striatum (p = 0.87) and nucleus accumbens (p = 0.84)), highlighting the near-complete rescue of TH signal in adult cKO mice.

To validate these findings using the TH biomarker, we immunostained brain sections for the dopamine transporter (DAT) as an independent marker of dopaminergic integrity (Fig. 2C). This analysis revealed a significant increase in DAT + signal in the striatum (p = 0.001) and nucleus accumbens (p = 0.023) of cKO mice injected with Fbxo7 virus at 4 weeks and harvested at 10 weeks as compared to uninjected cKO mice (Fig. 2D). These data demonstrate that re-introducing Fbxo7 at 4 weeks, before significant pathological changes are evident, provides protection against the loss of nigrostriatal terminals.

### Therapeutic Fbxo7 expression reverses established nigrostriatal pathology

**Fig. 3:**
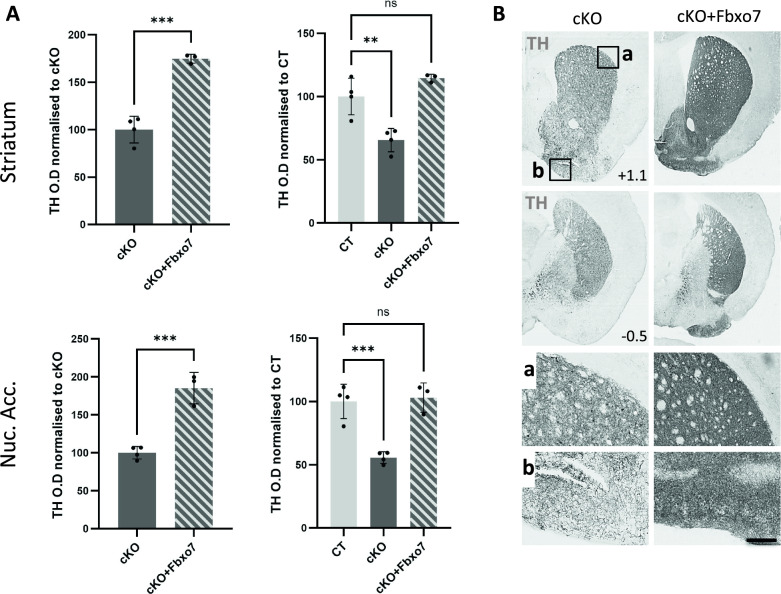
**A ** Images of TH-immunostained striatum at + 1.1 mm (top) and − 0.5 mm (bottom) relative to bregma. (**a**,**b **) Magnified boxed regions of striatum (a) and olfactory tubercle. **B ** OD quantification of TH + fibre density in CT and cKO mice at 15 weeks, uninjected or injected with Fbxo7 virus. Values are normalised to cKO or CT (as indicated on y axis) mean and shown as mean ± SEM with individual points. n = 3 or 4 per group. Statistical analysis was performed using a one-way ANOVA followed by Šídák's correction as a post-hoc test. Scale bar: 800 μm (top panels), 300 μm (**a**,**b**). *p < 0.05; ** *p*< 0.01; ***p < 0.001; ns, not significant

We next tested whether the delivery of Fbxo7 using rAAV could act as a therapeutic agent after significant loss of TH + staining is already apparent in the striatum. Based on our previous work showing a ~ 30–44% loss of TH immunoreactivity between 6 and 12 weeks [4], we chose 9 weeks of age as our intervention point. This is also the end of the period of postnatal maturation of the nigrostriatal circuitry, which occurs from E16 until 8–9 weeks, when mice are physically mature. As before, we allowed a 6-week recovery period prior to harvesting brains for analysis at 15 weeks. At 15 weeks, uninjected cKO mice showed the expected significant reduction in TH + staining, 34.5% (p = 0.0045) in the striatum and 44.4% (p = 0.001) in the nucleus accumbens (Fig. 3A), consistent with our previous report [4]. Remarkably, brains from cKO mice injected with the Fbxo7 rAAV showed robust TH staining in both regions (Fig. 3A). Compared to untreated cKO mice, this corresponded to a substantial increase to 174.8% in the striatum (p = 0.007) and 185.4% in the nucleus accumbens (p = 0.001), effectively restoring TH immunoreactivity to levels comparable to CT mice (striatum: 114.5%, p = 0.3; nucleus accumbens: 103.1%, p = 0.1) (Fig. 3B). These data demonstrate that Fbxo7 re-expression can rescue TH deficit back to control levels even after substantial pathology is established and after the postnatal maturation period. This suggests that the nigrostriatal terminals in this model were in a dysfunctional but salvageable state, arguing that Fbxo7-regulated pathways represent a potent therapeutic target for restoring neuronal integrity in the context of ongoing terminal loss or impairment.

## Discussion

Fbxo7 performs a wide range of functions essential to a variety of cell types, including neurons, glia, B and T lymphocytes, sperm and erythrocytes [4–7, 29–34]. It is critical for neuronal health, and mutations in the *FBXO7* gene drive a severe and progressive nigrostriatal degeneration that mimics PD pathology. The mechanistic basis for this neuropathology has been linked to its dual impact on mitochondrial homeostasis and proteasome dysfunction [5, 7, 11, 35–39]. Given its pleiotropic effects, a key question is whether targeting any of these downstream pathways is sufficient to correct the resulting deficits. A recent study addressed this, showing the suppression of mouse motor neuron pathology in an Fbxo7 null mouse by transgenic expression of a proteasome regulator PI31 [40]. PI31 is an Fbxo7 interacting protein, and the two are structurally related [11, 41–43]. This rescue demonstrates a clear epistatic relationship, establishing that PI31 acts downstream of Fbxo7 and that a significant portion of Fbxo7-driven neuropathology is mediated through the loss of PI31-dependent proteasome regulation. However, a striking observation in this study was that although there was a significant rescue of phenotypes, the transgenic expression of PI31 failed to rescue the lethality of an Fbxo7 null mutation strongly suggesting that Fbxo7 has additional functions that are essential for durable, long-term viability. This raised the critical question of whether restoring the Fbxo7 protein, rather than a single downstream effector, would rescue its neuropathological phenotypes. Our study demonstrates that Fbxo7 can prevent and even reverse established dopaminergic terminal deficiency in a progressive model of parkinsonism. Remarkably, the use of recombinant AAV (rAAV) to deliver Fbxo7 specifically to dopaminergic neurons prevented the significant reduction of striatal TH and DAT immunoreactivity in the striatum of younger mice and restored the density of TH + staining where the deficit had already occurred in older mice. Mice were injected at two points: at 4 weeks which is within the period of postnatal maturation, spanning from E16 to 8–9 weeks, and at 9 weeks, the end of that period, when mice are physically and sexually mature. One interpretation is that the re-expression of Fbxo7 at 4 weeks is a genetic rescue of defective development rather than pathological axonal loss, and that with injection of rAAV at 9 weeks, the maturation period is extended out to 15 weeks. However, we demonstrated here and in our previous study that development proceeded normally in cKO mice until 4 weeks. Significant pathological phenotypes only became apparent at 6 weeks and included a reduction in striatal dopamine which progressed to eventual DA neuronal loss in the SNpc at 20 weeks. Even if one proposes the later-stage rescue elicits an extension of the maturation period, it highlights a degree of reversibility of the phenotypes, and points to a window of plasticity of the nigrostriatal circuitry in adulthood. Our findings provide clear evidence that restoring Fbxo7-regulated pathways can counteract the emergence of early onset pathology and restore established dopaminergic terminal deficits. Future studies to treat mice at even later timepoints will determine the duration of that window. While our data show the recovery of dopaminergic markers to control levels, additional studies are needed to determine whether this represents a re-sprouting of terminal arbors or a biochemical rescue within a salvageable network of existing, but dysfunctional, terminals. Nevertheless, either mechanism would constitute a restoration with significant therapeutic implications. Future work will be aimed at dissecting the specific contributions that individual Fbxo7-regulated pathways have in achieving this re-expression of TH and DAT markers. The key question will be determining if targeting Fbxo7 and its downstream pathways can also prevent or restore the longer-term neuronal loss seen at 20 weeks and the behavioral deficits seen at 30 weeks that characterize this model of dopaminergic neurodegeneration.

A strength of our study was the use of a systemic, intravenous delivery of AAV9 serotype to achieve targeted gene expression in the midbrain. This approach enabled a less invasive route than direct stereotactic injection into neonate brains and the testing of different time points during disease progression in juvenile and adult animals. Our Cre-dependent expression cassette was designed to limit Fbxo7 expression to dopaminergic neurons, and our reporter data confirmed this strategy was successful, showing mRuby fluorescence within TH + neurons of the substantia nigra. Additional compelling evidence against a non-specific or off-target therapeutic mechanism comes from a more stringent empty vector (EV) control, where the EV group received a 3.7-fold higher viral load, yet exhibited no significant rescue. If the therapeutic effect were due to a general neuroinflammatory response or a trophic effect from low-level transduction of other cell types, e.g., glia, the higher-dose EV group should have shown an equal, if not greater, effect. This outcome strongly supports a cell-autonomous effect, driven by the specific biological action of Fbxo7 within dopaminergic neurons, rather than non-specific effects of the viral vector.

The landscape of rAAV gene therapy for PD patients has largely focused on two strategies: modulating circuit dysfunction, as with GAD delivery to the subthalamic nucleus, or providing general neuroprotection to slow disease progression, such as with growth factor delivery [44–46]. While these approaches have shown some clinical improvements, they are primarily symptomatic or aim to protect what remains of the nigrostriatal system. A more recent and promising frontier, exemplified by the ongoing clinical trial for GBA, is the direct correction of a specific genetic cause of PD [47]. Our findings with Fbxo7 place our work within this personalised medicine paradigm, and our study provides an important advance. We demonstrate that Fbxo7 expression can act prophylactically to preserve dopaminergic markers, and importantly, can restore these markers even after the deficit is already established, indicating that Fbxo7-dependent pathways are amenable to intervention. Given its dual role in mitochondrial and proteasomal regulation, therapeutic intervention with Fbxo7 may offer a strategy for restoring some aspects of dopaminergic terminal integrity in neurodegenerative diseases where these systems are impaired. This suggests we may expand beyond the current clinical goals of neuroprotection and slowing decline and pursue a strategy of phenotypic rescue and potential functional restoration.

Limitations of this study: While the results of this study provide an initial proof-of-concept for Fbxo7 gene therapy, its therapeutic efficacy was evaluated solely in an Fbxo7 cKO pathological model. For this to be taken forward, its effects in other pathological models and in a healthy, nigrostriatal system would be needed for wider applicability and future safety/toxicology profiling. To test for non-specific, vector-driven, neuroinflammatory or trophic effects, we included an EV control in our 10-week cohort. As a stringent stress-test, this EV group received a 3.7-fold higher viral titre than the Fbxo7-treated group. We observed a non-significant elevation in TH signal in these EV-treated mice, suggesting a minor response to the viral capsid. However, despite the much higher viral load, the EV treatment did not approach the highly significant, complete phenotypic rescue (restoring TH levels to > 110%) observed with the much lower-dose Fbxo7 vector. Because the 10-week cohort established that the viral capsid alone cannot account for the magnitude of the rescue, an EV control was not included at the 15-week time point. Although 10-week data support that the pathology reversal at 15 weeks is driven specifically by Fbxo7 expression, we cannot formally exclude a late-stage effect of the viral capsid. This will be addressed in future studies.

## Materials & Methods

### Mice

All experiments with mice were performed in accordance with the UK Animals (Scientific Procedures) Act 1986 and ARRIVE guidelines. Mice were housed in individually ventilated cages with unrestricted access to food and water, and a 12-h day-night cycle. All mice were bred as heterozygous crosses, and both male and female mice were used in experiments. *Dat*^*Cre*^* Fbxo7*^*fl/−*^ (cKO) and *Fbxo7*^*fl/−*^ (CT) mice were generated as previously described [4].

### Plasmids

pAAV-CAG-Flex-mRuby2-GSG-P2A-GCaMP6s-WPRE-pA was a gift from Tobias Bonhoeffer & Mark Huebener & Tobias Rose (Addgene plasmid # 68,717; http://n2t.net/addgene:68717; RRID: Addgene-68717). This construct was modified to remove GCaMP6s and replace it with mFbxo7. To achieve this, the MmFbxo7 gene (Image clone # 5,054,087) open reading frame was cloned in-frame with mRuby and P2A to generate an mRuby-P2A-Fbxo7 sequence. This sequence was then inserted in an antisense orientation relative to the chicken β-actin promoter and flanked by LoxP sites on both sides. Upon Cre recombinase expression, the transgene is flipped to the sense orientation, resulting in the activation of mRuby and Fbxo7 expression.

### rAAV production

The viral particles were produced by Penn Vector Core (Gene Therapy Program Perelman School of Medicine, University of Pennsylvania, USA). To generate the Empty Vector and Fbxo7 viruses, Penn Vector Core was provided with the plasmid DNA: AAV9-PHP.eB.CAG.DIO.P2A.mRuby (Empty Vector, EV) and AAV9-PHP.eB.CAG.DIO.FBXO7.P2A.mRuby (Fbxo7). Plasmid DNA was purified using an EndoFree Midi Prep Kit (Qiagen, Cat#12,362) to ensure low endotoxin levels, suitable for viral production. The titre of the viral preparations obtained was 5.5 × 10^13^ genome copies (GC)/mL for the EV virus and 7.52 × 10^12^ GC/mL for the Fbxo7 virus.

### rAAV injections

4- or 9-week-old *Dat*^*Cre*^* Fbxo7*^*fl/−*^ (cKO) or *Fbxo7*^*fl/−*^ (CT) mice were administered a single intravenous injection of an rAAV encoding an mRuby reporter gene and the murine Fbxo7 payload or an empty vector, containing only the mRuby reporter gene. In a total volume of 180 μL phosphate-buffered saline, the Fbxo7 group received 7.5 × 10^11^ GC of virus, while the EV virus group received 2.75 × 10^12^ GC of virus, approximately 3.7 times the amount of the Fbxo7 virus group. The increased titre injected into the EV group was considered in the Results (see Discussion). After the injection, mice were left to recover for 6 weeks before harvesting.

### Tissue processing

Brains were harvested at the age of 10 weeks or 15 weeks, as indicated. Mice were euthanised with a 0.3 mL intraperitoneal injection of pentobarbitone sodium, 200 mg/mL (Euthatal, Merial, UK). They were perfused transcardially with phosphate buffer saline (PBS) followed by ice-cold 4% paraformaldehyde in PBS (pH 7.4). Brains were removed and post-fixed in 4% PFA overnight before being transferred to 30% sucrose solution for cryoprotection.

### Immunohistochemistry

Perfused brains were sectioned coronally at 35 µm intervals using a sledge microtome (Leica, CM 1950). Sections were collected in 12 series and washed in 0.1 M phosphate-buffered saline before being incubated with primary and secondary antibodies (in 5% serum/0.05% Triton). Antibodies used were rabbit anti-Tyrosine Hydroxylase (TH) (Merck, ab152, 1:400), rabbit anti-dopamine transporter (DAT) (Proteintech, 22524–1-AP, 1:100), and secondary antibodies conjugated to Alexa Fluor 488 (Invitrogen, 1:400) were used for immunofluorescence detection. Sections were visualised by diaminobenzidine (SignalStain® DAB Substrate Kit; Cell Signaling Cat# 8059) according to the manufacturer’s protocol,) or by immunofluorescence (visualised using Alexa Fluor 488).

### Viral transduction efficiency

Viral transduction efficiency was quantified in the substantia nigra pars compacta (SNpc). Coronal midbrain sections were immunostained for TH and imaged under identical acquisition settings. For each brain, a defined region of interest within the SNpc was selected in two anatomically matched coronal sections at comparable rostro-caudal levels and was analysed.

All TH + neurons and mRuby + cells within the defined SNpc region were manually counted using QuPath software. Transduction efficiency was calculated as the percentage of TH + neurons expressing mRuby (mRuby + /TH +  × 100). All positive cells on tissue sections were counted for at least 3 samples/experimental condition.

### Optical density analysis

To determine the fibre density in the striatum, the mean optical intensity was measured from the TH- and DAT-positive stained sections. All DAB-stained slides were imaged using the Nanozoomer-XR slide scanner (Hamamatsu Photonics, Japan), at a resolution of 0.23 µm/pixel using a 40X objective. The areas of interest were the dorsal striatum and the nucleus accumbens. For the striatum, the measurements were made on 12 coronal sections, delineated as being between + 1.7 and -1.4 mm relative to bregma. The lining of the ventricular wall and external capsule represented the medial and dorsal/lateral borders of the defined area of interest in the striatum. The ventral limit of the striatum was a diagonal line passing above the anterior commissure, between the external capsule and the ventricular wall. For more posterior sections, the lateral aspect of the globus pallidus was used as the medial border of the striatum, and a horizontal line was made between the external capsule and the globus pallidus to outline the ventral border. Density measurements for the nucleus accumbens were made across 4 coronal sections, approximately + 1.7 to + 1.0 mm, relative to bregma. A diagonal line passing down through the anterior commissure, between the external capsule and the lowest portion of the ventricular wall, was used as the dorsal border and the lowest limits of the olfactory tubercle defined the ventral perimeter of the area we defined as the nucleus accumbens. TH staining along the lateral stripe of the striatum represented the lateral wall, and the TH + shell of the nucleus accumbens was used as the medial limits. For all DAB-based optical density measurements (TH and DAT), background correction was performed prior to normalisation. Optical density was measured in the defined region of interest and anatomical boundaries described above using ImageJ. Non-specific background was determined by measuring optical density in the corpus callosum and the white light background measured from the glass slide adjacent to the tissue section. Background values were subtracted from the corresponding regional measurements for each section.

Corrected optical density values were then normalised to the mean optical density of the control group by dividing each individual value by the control mean and expressing the result as percentage of control.

### Statistical analysis

Statistical analyses were performed on individual raw optical density (OD) values using GraphPad Prism software. For experiments including four independent groups (CT, cKO, cKO + EV, cKO + Fbxo7 virus), data were analysed using one-way ANOVA. Post-hoc pairwise comparisons were performed and corrected for multiple comparisons using Šídák's method, with the cKO or CT (as indicated) group used as the reference control. For comparisons between two groups only (cKO, cKO + Fbxo7 virus), unpaired two-tailed Student’s t-tests were performed.

For graphical presentation, values were normalised to the mean of the cKO or CT group (as indicated in the respective figure) and are shown as mean ± SEM with individual data points displayed. The number of animals per group (n = 3–5) is indicated in the corresponding figure legends. Statistical significance was set at *p* < 0.05 and is indicated as follows: ns, not significant (*p* ≥ 0.05); *p < 0.05; **p < 0.01; ***p < 0.001.

## Data Availability

All data generated or analysed during this study are included in this published article.
